# Spontaneous Headshake after a Kinematic Event (SHAAKE): Evaluating the Utility of a Potential New Sign in the Diagnosis of Concussion

**DOI:** 10.3390/diagnostics14202314

**Published:** 2024-10-17

**Authors:** Christopher J. Nowinski, Samantha C. Bureau, Hye Chang Rhim, Ross D. Zafonte, Robert C. Cantu, Daniel H. Daneshvar

**Affiliations:** 1 Concussion Legacy Foundation, Boston, MA 02115, USA; 2 Boston University Alzheimer’s Disease Research and CTE Centers, Boston University Chobanian & Avedisian School of Medicine, Boston, MA 02118, USA; 3 Department of Physical Medicine and Rehabilitation, Spaulding Rehabilitation Hospital, Harvard Medical School, Boston, MA 02129, USA; 4 School of Medicine, University of Missouri, Columbia, MO 65212, USA

**Keywords:** concussion, head injury, headshake after a kinematic event

## Abstract

**Background/Objectives**: Diagnosing concussions is problematic, in part due to the invisible nature of concussion symptoms, in addition to personal and interpersonal factors that influence symptom reporting. As a result, observable signs of concussion can ensure concussions are identified and appropriately treated. Here, we define a potential novel sign, the spontaneous headshake after a kinematic event (SHAAKE) and evaluate its utility in the diagnosis of concussion. **Methods**: A cross-sectional survey study of 347 athletes (age 27, IQR: 25–29; 47.6% female; highest level of play: college—46.1%, high school—41.2%) identified whether SHAAKE occurred, the reasons underlying SHAAKEs, and its utility for self-reported concussion. Sensitivity and positive predictive value were calculated across all sports and these parameters, as well as estimates for specificity and negative predictive value leveraging published helmet sensor data, were calculated for football players. **Results**: The median number of times participants reported SHAAKE was 5 (IQR: 3–10), with 4 (IQR: 2–7) associated with a self-reported concussion. Overall, 84.9% of participants reported concussion symptoms as the most common reason for their SHAAKEs. Across all sports, SHAAKE had a sensitivity of 49.6% and positive predictive value 72.4% for diagnosing concussion. In football players, SHAAKE had a sensitivity of 52.3%, estimated specificity of 99.9%, positive predictive value of 91.9%, and estimated negative predictive value of 99.5% for diagnosing self-reported concussion. **Conclusions**: These results demonstrate that nearly three-quarters of athletes reported a SHAAKE associated with a self-reported concussion, which supports the potential for SHAAKE to be used as a concussion screening tool.

## 1. Introduction

Concussion care has become one of the highest profile areas in clinical sports medicine over the last thirty years due to growing recognition of the risks associated with undiagnosed traumatic brain injuries [1]. The consequences of even delaying the diagnosis of concussion include prolonged recovery as well as higher rates of subsequent neurologic [1], and even orthopedic injury [2,3]. As a result, when a concussion is suspected, modern concussion protocols mandate immediate removal from play or immediate assessment [4,5,6,7]. Education initiatives have also aimed to promote the early identification of these injuries to improve triage for appropriate management [8,9,10,11,12].

The gold standard for concussion diagnosis remains clinical judgement by an appropriate health care practitioner [13]. Each concussion can present differently based on the specific characteristics of the injury and individual, which lead to differential neurotransmitter and metabolic cascades, resulting in a different series of possible blood flow changes, axonal injury, and neuroinflammation. To better reflect this heterogeneity, the Concussion in Sport group recently updated their sport-related concussion criteria to reflect that “symptoms and signs may present immediately, or evolve over minutes or hours, and commonly resolve within days, but may be prolonged” [14].

Multiple tools are used to assist clinicians in the diagnosis of concussion. A meta-analysis of three leading standardized diagnostic tools for concussion (ImPACT, King-Devick Test, and Sport Concussion Assessment Tool) alongside a grouped analysis of exams investigating signs and symptoms through different protocols, found that sensitivity ranged from 0.5 to 0.88 and specificity ranged from 0.8 to 0.88 [15]. Harmon and colleagues found the Standardized Assessment of Concussion, which primarily assesses cognition, had poor sensitivity; 45% of athletes with a concussion scored the same or better than their baseline score [16]. They also found that a self-reported symptom score increase of two points was associated with a sensitivity of 86%, a specificity of 80%, and a positive predictive value of 81%. However, in this study, only 34% of athletes were assessed within two hours of the impact that caused their concussion. Considering symptoms do not always present immediately, the extent to which self-reported symptoms are valuable in a sideline context is unclear.

Multiple major organizations have recently updated their concussion diagnostic criteria, and all now include recognition of observable signs of concussion as a pathway to diagnosis (Table 1). For example, the American College of Rehabilitation Medicine report states that one or more observable clinical signs of concussion, which cannot be better accounted for, and after a plausible mechanism of injury, is sufficient to diagnose concussion [17]. While the sign can be observed by a clinician, the ACRM criteria also consider “observable behaviors elicited through self-report” to qualify.

The predictive utility of observable possible signs of concussion is an area of growing scholarship. A retrospective video study of the updated National Football League (NFL) concussion protocol reported that the use of the visual signs checklist alone demonstrated “poor” ability to discriminate between individuals diagnosed with a concussion based on a full sideline assessment and those who were not diagnosed [21]. Specifically, in that study, two experts independently reviewed video of 251 plays where there was sufficient concern that a concussion occurred, which resulted in subsequent evaluation. On review of these videos, 211 resulted in a diagnosed concussion and 40 did not. For this subset of plays flagged due to concern for injury, the experts reported that the visual signs checklist had an overall sensitivity of 73% and specificity of 65%. The most sensitive was slow to get up at 66%, and the most specific were blank/vacant look and impact seizure, both of which at 100%.

A weakness of sensitivity and specificity calculations that limit analyses to videos flagged for potential concussions, is that these data necessarily only reflect the injuries that were ultimately diagnosed. Unfortunately, the majority of concussions are undiagnosed [22], potentially due to athlete knowledge, beliefs, and incentives [22,23,24,25,26]. Because the undiagnosed concussions are precisely the most difficult ones to identify, assessing the sensitivity and specificity of concussion signs based on retrospective video analysis of flagged plays biases towards the injuries easiest to identify, thereby artificially inflating observed sensitivity.

An analysis of eight concussion signs used in Australian Rules Football, again based on examination of videos flagged due to concussion concern, found that the sign “slow to get up” was the most sensitive (87%) but at the expense of specificity (19%). This study also revealed the subjectivity of many concussion signs, finding that inter-rater reliability for two major concussion signs, “loss of responsiveness” and “blank and vacant look”, was “fair” and did not reach statistical significance [27]. These signs serve as the basis of “red flags” and “features of a suspected concussion” in Australian Rules Football [28].

An analysis of six proposed international consensus signs in National Rugby League players addressed the concerns of bias due to video selection by examining “any in-game event wherein at least one player sustained a blunt trauma to the head and/or face via an external force.” For these analyses, six concussion signs demonstrated a “fair” reliability with an area under the receiver operating characteristics curve of 0.76. Blank/vacant look was the most sensitive sign at 54%, while tonic posturing was the most specific at 99% [29]. These signs have now been included in the concussion guidance for World Rugby [30].

Despite the wide range of sensitivities and specificities of existing concussion signs, there is increasing recognition of their importance to initiate a clinical evaluation and minimize the negative sequelae of continued play while concussed. This emphasis is most clearly highlighted by the addition of dedicated “concussion spotters” at games in the NFL, National Hockey League (NHL), Australian Rules Football, and World Rugby. Given the difficulties ascertaining symptoms in chaotic, in-game environments, with athlete incentives at times misaligned with athlete long-term health goals, recognizing additional signs of concussion is critical to identifying athletes who would benefit from additional concussion screening.

There is one potential sign of concussion that is widely used in multiple forms of entertainment—films [31], cartoons [32,33,34], and professional wrestling [35]—to indicate to the audience that a concussion has occurred, and yet it is not included in any major list of concussion signs, does not appear in the medical literature in any form, and does not have a medical name.

After a collision, individuals sometimes rapidly shake their head back and forth, which we have termed the spontaneous headshake after a kinematic event (SHAAKE). The SHAAKE appears to be voluntary and generally occurs seconds to minutes after the impact. We hypothesize that the SHAAKE is associated with self-reported concussion symptoms, with high sensitivity and specificity for self-reported concussion. The aim of the present study was to determine how common athletes exhibited this SHAAKE, identify the reasons why they made the SHAAKE, and evaluate the sensitivity, specificity, and positive predictive value of its association with self-reported concussions.

## 2. Materials and Methods

### 2.1. Sample

Survey participants were recruited from the international clinical research registry of the Concussion Legacy Foundation, a 501 (c) (3) public charity based in the United States with chapters in Canada, Australia, and the United Kingdom. Individuals must be at least 18 years of age to join the registry. Inclusion criteria included: playing sports at any level, and age 30 or under. The study was limited to younger athletes to limit recall bias and to minimize the risk of the data being influenced by outdated definitions of concussion, given changes due to rapid advances in the scientific understanding of concussion over the past 20 years [36,37]. In addition, surveying younger athletes increased the likelihood that participants had received formal concussion education due to the proliferation of state concussion laws requiring education of athletes that were put in place between 2009 and 2012 [38]. There were no additional inclusion or exclusion criteria. Our study was conducted in accordance with ethical standards and was granted exemption from Institutional Review Board (IRB) review (2024P000442).

### 2.2. Procedure

**Figure 1:**
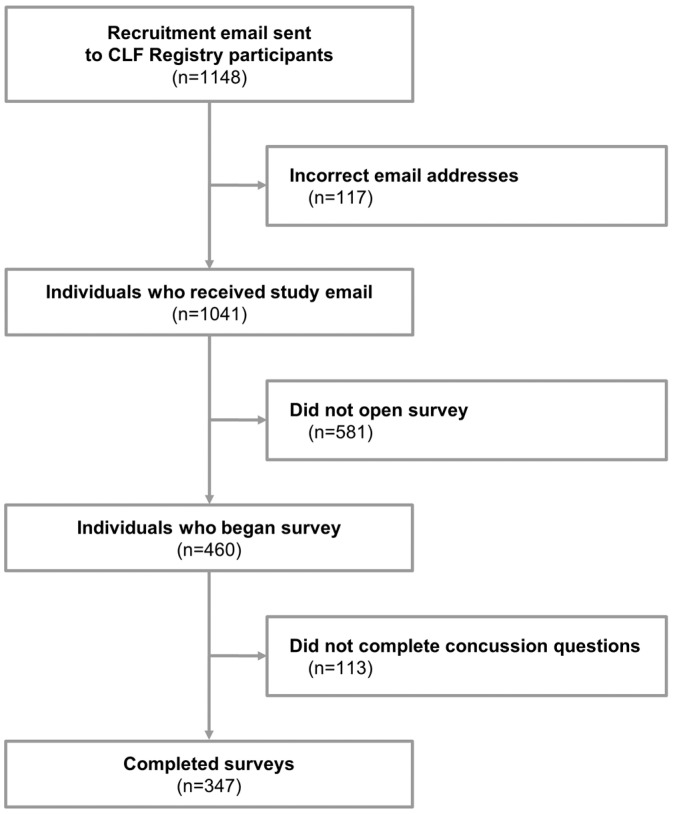
Flow chart of inclusion into study. CLF: Concussion Legacy Foundation.

A cross-sectional study design was used to acquire data. Surveys were emailed to participants in June and July 2024. If the initial email did not elicit a response, a second email was sent within four weeks of the initial email. In total, 1041 individuals were sent surveys (Figure 1), and 460 surveys were started. Surveys that were stopped prior to the concussion questions were considered incomplete (*n* = 113), resulting in a total of 347 completed surveys (response rate: 33.3%).

### 2.3. Survey

Participants were asked their age, gender, race and ethnicity, highest level of sports participation, whether they served in the military, the region of the world in which they reside, and sports they played competitively (Supplementary Materials Table S1 for complete survey). To minimize bias, participants were not asked any questions pertaining to concussion prior to being asked about whether they had experienced any SHAAKEs. To best depict the intended movement, five video examples were provided demonstrating a sports impact followed within minutes by a rapid shaking of the head laterally back and forth at least one full cycle at a rate between 2–8 Hz. The videos were chosen to be representative across gender and sport, and included male and female athletes playing American football, basketball, baseball, and soccer, chosen through consensus and reviewed by a team of physicians and neuroscientists (CJN, SCB, RCC, DHD). The video is publicly available here: https://youtu.be/J3Bu9_lpGXs.

After viewing the video, participants were asked “Do you remember ever making this type of head motion after a collision?” If they answered yes, the participants were shown a list of thirteen possible reasons for making that motion, including symptoms associated with concussion (changes in vision, dizziness, auditory changes) and symptoms not associated with concussion (scalp pain, a feeling of chills, emotional reaction to the preceding event), and were asked to select all that applied. An “other” category was included inviting participants to suggest other reasons they made the movement after a collision. If they answered yes to changes in vision or auditory changes, additional questions asked them to describe the type of vision or auditory change (e.g., double vision, blurred vision). Participants were then asked which of the thirteen reasons was the single most common reason they displayed this movement. They were then asked to estimate how many times they had rapidly shaken their heads following a collision.

Participants were then provided a standardized definition of concussion [36], and asked how many concussions they had experienced, followed by whether they had experienced a SHAAKE after a concussion and, for those that had, how many in total were formally diagnosed, and what symptoms were associated with each. Participants were then asked when they had their most recent concussion and to estimate what proportion of their concussions were from sports or non-sport activities.

### 2.4. Statistical Analysis

Because continuous variables were not normally distributed, medians and interquartile ranges were provided. To evaluate the utility of SHAAKE for diagnosing concussions: true positives were defined as the number of SHAAKEs a respondent reported because of a concussion; false positives were the total number of SHAAKEs a respondent reported minus the total number of SHAAKEs that occurred because of a concussion; false negatives were the total number of concussions reported minus the total number of SHAAKEs that occurred because of concussion; and true negatives were defined as the total number of times an athlete was hit in the head without a SHAAKE or concussion.

For true negatives, the total number of times an athlete was hit in the head could not be directly ascertained but could be extrapolated based on previously published sport-specific data. Unfortunately, the total number of times an athlete experiences hits to the head is not known for most sports. Football helmet sensor studies published through 2021 were recently aggregated across all positions at the youth, high school, and collegiate levels, to generate a mean number of hits greater than 10 g of 532 hits per season at the high school and collegiate level [39]; high school and college were chosen as the vast majority of athletes indicated these to be their highest level of football play (*n* = 112, 92.6%). Therefore, true negatives were only estimated for football players, which was calculated as the number of years an athlete reported playing football times the average number of hits a football player experiences based on helmet sensor data (532 hits/season), minus the true positives, false positives, and false negatives.

Secondary analyses explored additional differences between football and soccer players using chi-square (or Fisher’s exact when more than 20% of cells had an expected count of five or fewer) and *t*-tests (or Wilcoxon’s rank sum when non-normally distributed); athletes who played both football and soccer were categorized based on which sport they played the most seasons. Secondary analyses also explored whether there were relationships between the percentage of concussions caused by sport, or time since most recent concussion (each in separate models as possible predictors), and concussion number, or number of SHAAKEs due to a concussion (each in separate models as possible outcomes), using logistic regression. All analyses were performed using R version 4.3.2.

## 3. Results

### 3.1. Participants

In total, 347 participants completed the survey (Figure 1). The median age of survey participants was 27 (IQR 25–29), about half (47.6%) identified as female, and 92.2% described themselves as White or Caucasian. Nearly all respondents were from North America (79.3%), either the United States (64.6%) or Canada (14.7%). Their highest level of sports played was most frequently collegiate (46.1%), followed by high school (41.2%) and semi-professional (6.6%). The ten most popular sports played, in order of participation, were football, soccer, basketball, track and field, ice hockey, rugby, baseball, lacrosse, wrestling, and volleyball. Twelve (3.5%) served in the military. Full descriptive data for the study sample are provided in Table 2.

### 3.2. Concussion Experience

Participants reported a high frequency of concussion, with 98.6% saying they had at least one concussion after being provided a modern definition. The mean number of self-reported concussions was 23.8 ± 131.5, and the median was 6 (IQR: 4–11). The mean number of diagnosed concussions reported was 3.8 ± 3.2, with a median of 3 (IQR: 2–5). Participants reported 90% of their concussions occurred while playing sports, with 21.4% reporting their most recent concussion occurred within the last year, 40.0% between one and five years ago, 35.6% between five years and ten years ago, and 6.8% more than ten years ago (Table 3).

### 3.3. SHAAKE Experience and Association with Concussion

The majority (68.9%) of participants reported rapidly shaking their head after a collision at least one time. After being provided with a modern concussion definition, 64.3% of all participants reported making this movement in association with a concussion; 93.3% of participants who reported a SHAAKE associated it at least one time with a concussion. The mean number of times participants reported this movement after a collision was 23.3 ± 80.7 and the median was 5 (IQR: 3–10). The mean number of times participants reported this movement due to a concussion was 18.2 ± 136.0, with a median of 4 (IQR: 2–7; Table 4).

The reasons that participants reported for why they exhibited the SHAAKE are provided in Table 5; the top three were disorientation or confusion (71.7%), dizziness (54.0%), and a feeling like they needed to jumpstart their brain (52.3%). Overall, 84.9% of participants reported that the most common reason they exhibited a SHAAKE was due to a symptom of concussion; only 15.1% reported it was due to non-concussion-related reasons. When asked for all the reasons they had ever exhibited the SHAAKE, the 239 participants who had exhibited the SHAAKE reported 1410 symptoms, an average of 5.90 symptoms per participant. Concussion symptoms comprised 84.8% of the 1410 symptoms.

The list of thirteen reasons why an athlete might SHAAKE proved to be comprehensive, with at least one participant reporting a SHAAKE for each reason, and only eight providing other reasons that were not on the list (see Supplementary Materials Table S2 for the eight reasons). While the list was not proposed to be exhaustive, the fact that only eight athletes provided “other” reasons, out of 1410 explanations endorsed, suggests all the major reasons why an athlete would SHAAKE were captured.

For athletes across all sports, the total number of SHAAKEs that were reported because of concussions (true positives) were 4035. The total number of SHAAKEs that were reported overall were 5573, resulting in 1538 false positives. The total number of concussions reported was 8136, resulting in 4101 false negatives. As a result, for the diagnosis of concussion across all sports, SHAAKE had a sensitivity of 49.6% and a positive predictive value 72.4%.

Specifically for the 109 football players, the total number of SHAAKEs that occurred because of concussions (true positives) was 2851. The total number of SHAAKEs reported was 3102 resulting in 251 false positives. There were 5453 total concussions reported resulting in 2602 false negatives. Football players reported a cumulative 950 years of football played, which resulted in 505,400 estimated head impacts and 499,696 true negatives. As a result, for the diagnosis of concussion in football players, SHAAKE had a sensitivity of 52.3%, estimated specificity of 99.9%, positive predictive value of 91.9%, and estimated negative predictive value of 99.5%.

To examine if there were sport-related differences in the presentation of SHAAKE and relationship with concussion, secondary analyses examined potential differences between the top two most common sports: football and soccer. There were significant differences between sports in gender and seasons of play (Table 2), concussion number, time since last concussion, and percentage of sports concussions (Table 3) and number of SHAAKEs (Table 4), but no differences in the rate with which SHAAKE was associated with concussion (Table 4) or the reasons for the SHAAKE with or without concussion (Table 5, all *p* > 0.05). Additional secondary analyses found that neither the percentage of concussions caused by sport, nor the time since most recent concussion, were associated the number of concussions (all *p* > 0.05) or SHAAKEs due to a concussion (all *p* > 0.05).

## 4. Discussion

We surveyed current and former young adult athletes to understand the relationship between a specific type of headshake often observed after a collision and concussion symptoms. These data provide evidence that this movement is both familiar to athletes, with seven out of every ten athletes recalling making this movement after a collision, and closely tied to concussion symptoms, with nearly 95% having made the movement at least once for symptoms of a suspected concussion.

There was a strong relationship between SHAAKE and concussion symptoms. To the best of our knowledge, a prior description of this clinical entity does not appear in the literature. The closest description we found was the observation of a “transient, isolated head tremor” being proposed as an early manifestation of essential tremor, unrelated to a kinematic event [40]. However, SHAAKE is widely used in multiple forms of entertainment—films [31], cartoons [32,33,34], and professional wrestling [35]—to indicate to the audience that a concussion has occurred.

Our data demonstrate that for self-reported suspected concussion, SHAAKE has a positive predictive value of 72.4%; nearly three-quarters of the time athletes reported a SHAAKE, they said it was associated with a concussion. Using estimates of head impacts based on helmet sensor data, we were able to calculate additional parameters for football players, over 90% of the times football players reported a SHAAKE, they said it was associated with a concussion. Unsurprisingly, the majority of football collisions were not associated with concussions or SHAAKEs, resulting in a very high estimated specificity and estimated negative predictive value for the use of SHAAKE in the diagnosis of concussion for football.

**Figure 2:**
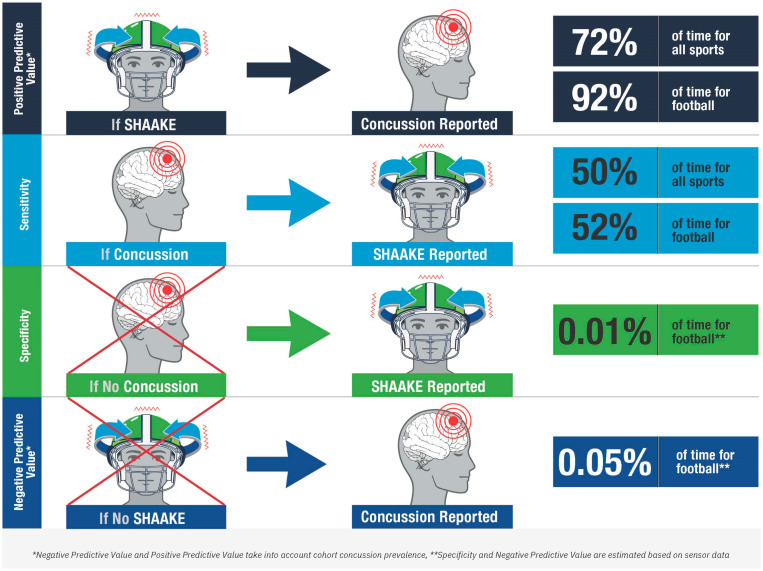
Accuracy of the spontaneous headshake after a kinematic event (SHAAKE) in the diagnosis of concussion.

While caution is needed when interpreting our sensitivity and specificity derived from self-reported data, these measures, in particular the high positive predictive values across all sports and in football specifically, place SHAAKE among the most predictive signs of concussion (see Figure 2). With a sensitivity of 50%, it is nearly as sensitive as the most sensitive sign in the National Rugby League study (blank/vacant look, 54%; notably the one study to examine all head impacts rather than just the concerning ones) and NFL study (slow to get up, 66%). Of note, these relatively lower sensitivities across all concussion signs are unsurprising; the majority of well-established concussion signs (e.g., emesis, loss of consciousness, seizures) occur relatively infrequently after a concussion occurs (e.g., the vast majority of concussion do not result in loss of consciousness). As such, a sensitivity of 50%, meaning that SHAAKE occurs around half the time a concussion happens, actually indicates a strong relationship between SHAAKE and concussion, compared to most concussion signs. With an estimated specificity of 99.9% among football players, it is also among the most specific signs of concussion, similar to loss of consciousness, impact seizure, or tonic posturing.

The relatively high number of false negatives was expected, as it is unlikely a concussion sign that was consistently associated with concussion would have remained hidden for so long. The modest number of false positives suggests that it would not be appropriate to include SHAAKE among, for example, the NFL’s “No-Go” concussion signs that require immediate and permanent removal from the game. Instead, SHAAKE may best fit among potential signs of concussion that suggest consideration of removal from play for assessment, similar to “slow to get up” or “clutching of the head after contact.”

In a chaotic sideline environment, it can be difficult to reliably screen athletes for further evaluation. Given the negative sequelae associated with undiagnosed concussions, including the increased risk of subsequent injury or catastrophic brain injury from second-impact syndrome, any additional tool to help identify athletes for further concussion evaluation would provide a valuable contribution to athlete health [1]. Furthermore, if SHAAKE is used only to prompt a sideline evaluation, rather than be used to independently diagnose concussion, there is little harm associated with additional screening due to false positives; briefly assessing an athlete for further indications of concussion on the sideline is relatively benign, particularly given that nearly 70% of concussions occur in practice [41]. In addition, if athletes become aware that a SHAAKE related to causes other than concussion (e.g., emotional reactions to a preceding play) may lead to a sideline evaluation, their behavior may change, and athletes may be less likely to SHAAKE in the absence of concussion symptoms.

If SHAAKE had been included among possible signs of concussion in the NFL concussion protocol in 2022, it may have changed the diagnosis in one of the most scrutinized and controversial concussion evaluations in the history of sports. In a game against the Buffalo Bills on 25 September 2022, Miami Dolphins quarterback Tua Tagovailoa fell backwards and hit his head on the ground. Per reports, “after his head slammed into the turf, Tagovailoa got up and staggered, repeatedly shaking his head [displaying a SHAAKE] and failing to stay on his feet” [42]. A sideline doctor concluded that his fall was not due to ataxia, and thus evidence of a concussion, but rather due to a previous injury to his back. Even with the benefit of hindsight, in the days following the game, his imbalance after the collision was attributed to a previous back injury, rather than ataxia caused by a concussion. The SHAAKE he exhibited was not considered a possible sign of concussion. Perhaps if the doctor had access to these data, which demonstrate that SHAAKE has a positive predictive value for concussion of over 90% in football players, the doctor may have come to a different clinical conclusion and not cleared Tagovailoa. In this instance, the SHAAKE he exhibited would be difficult to attribute to a prior back injury. Any of the doctors that saw Tua, both on the field and in the subsequent days, may have reconsidered and, based on this additional evidence, determined that a concussion diagnosis would more appropriately cover both his imbalance and his SHAAKE. The presence of more than one observable concussion sign, which each result from impairments to different neurologic domains, increases the likelihood that a concussion occurred, by decreasing the likelihood of alternate explanations. Had a concussion been diagnosed, Tagovailoa would not have played in a game four days later, during which he lost consciousness following a concussion and displayed fencing response, before being hospitalized [43].

Additional work is needed to clarify the parameters of the SHAAKE. Based on our clinical experience and video review of this movement, SHAAKE is usually initiated within seconds or minutes of an impact to the head or body but can occur at any point concussion symptoms remain. SHAAKE occurs with a lateral rotation side to side at a rate of 2 Hz to 8 Hz, typically lasts less than two seconds, and it is not directed at another person as a form of communication (e.g., saying “no”). Additional video review and clinical assessment would help to further define this process.

As self-reported concussion symptoms remain the gold standard for concussion diagnosis, and athletes have significant personal and interpersonal incentives to not report concussion symptoms during the season, we believe these results may be more robust than previous studies, which used video selected specifically due to some exogenous concern that a concussion occurred in their sensitivity and specificity analyses. However, the validation of these findings with real-time prospective studies with video monitoring would further support the use of SHAAKE in concussion diagnostics. A severe limitation of future prospective studies of SHAAKE including video monitoring or clinical observation studies is that video is usually limited to the field of play, and medical personnel are not always looking at athletes once they leave the field, and SHAAKE may occur on the sideline, in the locker room, or somewhere else that cannot be observed.

Strengths of this study include sampling across sports, with less than one-third from football, across gender, with nearly one-half women, and across levels of play, ranging from high school through professional play. Additionally, by including younger participants, the effect of changing concussion definitions, athletic era, or years since play, are minimized. Prior to 2007, when concussions became part of a national conversation in the United States, many athletes and doctors believed that loss of consciousness was required to diagnose concussion. The individuals studied played sports during a period when modern definitions of concussions were used and concussion education was often mandatory. Former high school, college, and professional athletes under the age of 30 would have been more likely to play sports using a modern definition of concussion.

### Limitations

This study has several limitations. The study relied on self-reported data from respondents, which may introduce the potential for recall bias. However, we only included younger athletes to minimize such bias. Furthermore, the retrospective nature of this study leads to potential response bias; however, the fact that there was no relationship between time since last concussion and either the number of concussion symptoms, or the number of SHAAKEs associated with a concussion, indicates that recency was not a significant factor in these findings. We did not ask about preexisting neck injuries and how they might impact an athlete’s ability or willingness to SHAAKE and encourage subsequent studies to include this question. However, it is unlikely that athletes who cannot quickly shake their head back and forth are participating in competitive sports. Additionally, we did not have detailed information describing how many concussions were associated with each particular activity; the fact that the percentage of concussions associated with sport was also not significantly associated with either the number of concussion symptoms, or the number of SHAAKEs associated with a concussion, suggests that this was not a major driver of differences amongst this cohort of athletes. Participants were primarily from the United States and Canada, and it is not clear how the presentation of and reasons for SHAAKE may vary by region or country. Only 10% of participants were non-White; additional analyses are needed to explore whether there are any differences by race or ethnicity. We did not explore how the culture of injuries within each sport may impact SHAAKE. For example, the clinical value of SHAAKE could be different in international professional soccer where athletes are known to exaggerate injuries. Regarding the estimates for football based on helmet sensor data, these analyses included all measured kinematic events as a spontaneous headshake after a kinematic event necessarily occurs after a kinematic event, and concussions can and do occur across this range of kinematic events. However, at different thresholds (e.g., *g*-forces) of kinematic events, SHAAKE will show different predictive accuracy. Future studies should explore how the severity of symptoms relates to SHAAKE, how the time from the collision to the presentation of the movement influences sensitivity and specificity, as well as the outer limits of the time between collision to presentation to be considered as SHAAKE. This study should also be replicated, and prospective studies are recommended to validate the findings. Video analyses of kinematic events with contemporaneous evaluation for concussion would further validate SHAAKE as a concussion sign. Inter-rater and intra-rater reliability for recognizing SHAAKE based on this proposed definition would also be ascertained in prospective studies.

## 5. Conclusions

The rapid shaking of the head back and forth after a collision is a possible sign of concussion previously undescribed in the medical literature. This study provides preliminary evidence that this movement has high sensitivity, specificity (in football players), and positive predictive value based on the self-reported data. We have given this previously undescribed clinical entity a name: spontaneous headshake after a kinematic event, or SHAAKE. Despite the presence of SHAAKEs in movies, television, and cartoons for decades, this lay understanding of injury has not been integrated into formal concussion screening metrics. Because of this history and strong preliminary evidence, we suggest immediately adding SHAAKE to existing lists of potential signs of concussion and in sports concussion protocols and encourage sports organizations to evaluate SHAAKE across different populations using different methodologies.

That nearly three-quarters of SHAAKEs were associated with self-reported concussion, as well as the high sensitivity of SHAAKE amongst all athletes and the high specificity of SHAAKE for self-reported concussion in football players, supports its potential as a new concussion sign in the critical practice of recognizing and diagnosing concussion in athletes.

## Figures and Tables

**Table 1:** Signs of concussion provided by major health organizations, concussion guidelines, and sports organizations. U.S.: United States.

United States Centers for Disease Control and Prevention [18]	American Congress of Rehabilitation Medicine [17]	Concussion in Sport Group [19]	National Football League [20]
Cannot recall events prior to or after a hit or fall. Appears dazed or stunned. Forgets an instruction; is confused about an assignment or position; or is unsure of the game, score, or opponent. Moves clumsily. Answers questions slowly. Loses consciousness (even briefly). Shows mood, behavior, or personality changes.	The injury event causes an acute physiological disruption of brain function, as manifested by one or more of the clinical signs listed below. Loss of consciousness immediately following injury (e.g., no protective action taken on falling after impact or lying motion less and unresponsive). Alteration of mental status immediately following the injury (or upon regaining consciousness), evidenced by reduced responsiveness or inappropriate responses to external stimuli; slowness to respond to questions or instructions; agitated behavior; inability to follow two-part commands; or disorientation to time, place, or situation. Complete or partial amnesia for events immediately following the injury (or after regaining consciousness). If post-traumatic amnesia cannot be reliably assessed (e.g., due to polytrauma or sedating analgesics), retrograde amnesia (i.e., a gap in memory for events immediately preceding the injury) can be used as a replacement for this criterion. Other acute neurologic sign(s) (e.g., observed motor incoordination upon standing, seizure, or tonic posturing immediately following injury).	Lying motionless on playing surface. Falling unprotected to the surface. Balance/gait difficulties, motor incoordination, ataxia, stumbling, slow/laboured movements. Disorientation or confusion, staring or limited responsiveness, or an inability to respond appropriately to questions. Blank or vacant look. Facial injury after head trauma. Impact seizure. High-risk mechanism of injury (sport dependent).	National Football League no-go Loss of consciousness (including Impact Seizure and/or “fencing posture”). Ataxia (abnormality of balance/stability, motor coordination, or dysfunctional speech). Confusion. Amnesia. Potential concussion signs (observable) may include Any loss of consciousness. Impact seizure or “fencing” posture. Motor coordination/balance problems of neurologic etiology (stumbles, trips/falls, slow/labored movement). Blank or vacant look. Disorientation (e.g., unsure of where he is on the field or location of bench). Behavior change (aggressive, agitated, atypically subdued, unusually emotional or frightened, etc.). Amnesia, either anterograde or retrograde. Clutching of the head after contact. Visible facial injury in combination with any of the above.

**Table 2:** Descriptive data for all participants, as well as for soccer and football players.

		All Sports (*n* = 347)	Soccer (*n* = 99)	Football (*n* = 109)	*p*-Value *
**Age**		27 (IQR 25–29)	26 (IQR 23–29)	28 (IQR 26–29)	**<.001**
**Gender**					**<.001**
	Male	167 (48.1%)	21 (21.2%)	103 (94.5%)	
	Female	165 (47.6%)	72 (72.7%)	4 (3.7%)	
	Non-binary	12 (3.5%)	5 (5.1%)	0 (0%)	
	Transgender female	3 (0.9%)	1 (1.0%)	2 (1.8%)	
**Race and Ethnicity**					>.05
	American Indian or Alaska Native	13 (3.7%)	3 (3.0%)	5 (4.1%)	
	Asian	19 (5.5%)	5 (5.1%)	3 (2.8%)	
	Black or African American	13 (3.7%)	3 (3.0%)	8 (7.3%)	
	Hispanic or Latino or Spanish Origin	14 (4.0%)	8 (8.1%)	3 (2.8%)	
	White or Caucasian	320 (92.2%)	91 (91.9%)	100 (91.7%)	
**Highest Level of Sport Participation**					.19
	Youth	1 (0.3%)	1 (1.0%)	0 (0%)	
	High school/secondary school	143 (41.2%)	43 (43.4%)	47 (43.1%)	
	College	160 (46.1%)	44 (44.4%)	56 (51.4%)	
	Semi-professional	23 (6.6%)	3 (3.0%)	4 (3.7%)	
	Professional	11 (3.2%)	3 (3.0%)	2 (1.8%)	
	Other	9 (2.59%)	5 (5.1%)	0 (0%)	
**Number of Seasons**		8 (IQR 6–12)	12 (IQR 6–16)	8 (IQR 5–12)	**<.001**
**Military**		12 (3.5%)	2 (2.0%)	6 (5.5%)	.28
	Years of Military	6.8 ± 3.0	10 ± 4.2	5.8 ± 3.1	.17
**Geographical Distribution**					.72
	North America (Canada)	51 (14.7%)	10 (10.1%)	12 (11.0%)	
	North America (USA)	224 (64.6%)	69 (69.7%)	73 (66.1%)	
	Central America	67 (19.3%)	18 (18.2%)	23 (21.1%)	
	Europe (Northern)	1 (0.3%)	0 (0%)	0 (0%)	
	Europe (Western)	2 (0.29%)	1 (1.0%)	1 (0.9%)	
	Asia (Eastern)	1 (0.3%)	0 (0%)	0 (0%)	
	Other	1 (0.3%)	1 (1.0%)	0 (0%)	

* *p*-values are provided to assess differences between soccer and football players. For categorical variables, chi-square was performed except where Fisher’s exact test was more appropriate. For continuous variables, Wilcoxon’s rank sum tests were performed because data were non-normally distributed. IQR: interquartile range; USA: United States of America.

**Table 3:** Concussions experienced for all participants, as well as for soccer and football players.

		All Sports (*n* = 347)	Soccer (*n* = 99)	Football (*n* = 109)	*p*-Value *
**Ever had a concussion**		342 (98.6%)	99 (100%)	107 (98.2%)	.50
	Number of concussions	6 (IQR 4–11)	6 (IQR 4–12)	8 (IQR 5–15)	**.03**
	Number of diagnosed concussions	3 (IQR 2–5)	4 (IQR 2–6)	2 (IQR 2–4)	**.01**
**Most recent concussion timing**					**.003**
	Within the past week	1 (0.3%)	0	0	
	Between one week and one month ago	5 (1.5%)	4 (4.1%)	0	
	Between one month and six months age	22 (6.5%)	6 (6.1%)	9 (8.4%)	
	Between six months and one year ago	32 (9.4%)	15 (15.3%)	3 (2.8%)	
	Between one and five years ago	136 (40%)	37 (37.8%)	40 (37.4%)	
	Between five and ten years ago	121 (35.6%)	32 (32.7%)	431 (40.2%)	
	Between ten and twenty years ago	23 (6.8%)	4 (4.1%)	12 (11.2%)	
**Percent of concussions from sports**		90% (IQR 75–100)	84% (IQR 65–100)	100% (IQR 90–100)	**<.001**

* *p*-values are provided to assess differences between soccer and football players. For categorical variables, chi-square was performed except where Fisher’s exact test was more appropriate. For continuous variables, Wilcoxon’s rank sum tests were performed because data were non-normally distributed. IQR: interquartile range.

**Table 4:** Data pertaining to spontaneous headshake after a kinematic event and relationship to concussion for all participants, as well as for soccer and football players.

		All Sports (*n* = 347)	Soccer (*n* = 99)	Football (*n* = 109)	*p*-Value *
**Ever had a SHAAKE**		239 (68.9%)	71 (71.7%)	85 (78.0%)	.30
	Number of SHAAKEs	5 (IQR 3–10)	4 (IQR 3–6)	10 (IQR 5–20)	**<.001**
**Ever had a concussion**		342 (98.6%)	99 (100%)	107 (98.2%)	.50
**Ever had a SHAAKE because of a concussion**		223 (64.3%)	64 (64.6%)	80 (73.4%)	.11
	Number of SHAAKEs because of a concussion	4 (IQR 2–7)	3 (IQR 2–6)	5 (IQR 3–10)	**.01**
	Number of SHAAKEs with a undiagnosed concussions	1 (IQR 0–4)	1 (IQR 0–2)	3 (IQR 1–6.25)	.25

* *p*-values are provided to assess differences between soccer and football players. For categorical variables, chi-square was performed except where Fisher’s exact test was more appropriate. For continuous variables, Wilcoxon’s rank sum tests were performed because data were non-normally distributed. IQR: interquartile range; SHAAKE: spontaneous headshake after a kinematic event.

**Table 5:** Reasons why athletes had a spontaneous headshake after a kinematic event for all participants, as well as for soccer and football players.

				All Sports (*n* = 239)	Soccer (*n* = 71)	Football (*n* = 85)	*p*-Value *
**Reasons for SHAAKE** (**select all**)							.81
	Neck Pain			48 (20.1%)	15 (21.1%)	15 (17.6%)	
	A feeling of a change in temperature or chills			26 (10.9%)	7 (9.9%)	11 (12.9%)	
	Emotional reaction to preceding event			95 (39.7%)	27 (38.0%)	25 (29.4%)	
	Pain to your face, scalp, or other part of your head that was not a headache			46 (19.2%)	18 (25.4%)	14 (16.5%)	
	Changes in your vision			109 (45.6%)	36 (50.7%)	42 (49.4%)	.97
		Double vision		10 (14.1%)	14 (16.5%)	14 (16.5%)	
		Blurred vision		25 (35.2%)	31 (36.5%)	31 (36.5%)	
		Trouble focusing		22 (31.0%)	26 (30.6%)	26 (30.6%)	
		Changes in colors perceived		6 (8.5%)	6 (7.1%)	6 (7.1%)	
		Other		3 (4.2%)	4 (4.7%)	4 (4.7%)	
		Double vision		1 (1.4%)	3 (3.5%)	3 (3.5%)	
	Auditory changes			49 (20.5%)	15 (21.1%)	19 (22.4%)	.95
		Ringing in your ears		13 (18.3%)	17 (20.0%)	17 (20.0%)	
		Deafness or impaired ability to hear sounds		3 (4.2%)	3 (3.5%)	3 (3.5%)	
		Trouble recognizing sounds		7 (9.9%)	8 (9.4%)	8 (9.4%)	
	Dizziness			129 (54.0%)	30 (42.3%)	55 (64.7%)	
	Impaired ability to balance			71 (29.7%)	22 (31.0%)	30 (35.3%)	
	Changes to your perception of your body’s positioning or location in space (proprioception)			101 (42.3%)	28 (39.4%)	40 (47.1%)	
	Headache			71 (29.7%)	18 (25.4%)	28 (32.9%)	
	Disorientation or confusion			170 (71.1%)	47 (66.2%)	61 (71.8%)	
	Unable to keep your train of thought/inability to think clearly			73 (30.5%)	23 (32.4%)	32 (37.6%)	
	A feeling like you needed to jumpstart your brain			125 (52.3%)	40 (56.3%)	41 (48.2%)	
	Other			8 (3.3%)	2 (2.8%)	3 (3.5%)	
**Reasons for SHAAKE (most common)**							.62
	Neck pain			6 (2.5%)	2 (2.8%)	4 (4.7%)	
	A feeling of a change in temperature or chills			1 (0.4%)	0 (0%)	0 (0%)	
	Emotional reaction to preceding event			18 (7.5%)	6 (8.5%)	2 (2.4%)	
	Pain to your face, scalp, or other part of your head that was not a headache			9 (3.8%)	3 (4.2%)	3 (3.5%)	
	Changes in your vision			15 (6.3%)	7 (5.6%)	4 (4.7%)	.64
			Double vision	3 (20%)	1 (14.3%)	2 (50%)	
			Blurred vision	6 (40%)	3 (42.9%)	1 (25%)	
			Trouble focusing	2 (13.3%)	0	0	
			Changes in colors perceived	2 (13.3%)	1 (14.3%)	1 (25%)	
			Other	2 (13.3%)	2 (28.6%)	0	
	Auditory changes			4 (1.7%)	0 (0%)	3 (3.5%)	
			Trouble recognizing sounds	4 (100%)	0	3 (100%)	
	Dizziness			19 (8.0%)	4 (5.6%)	7 (8.2%)	
	Impaired ability to balance			4 (1.7%)	1 (1.4%)	2 (2.4%)	
	Changes to your perception of space or perception of your body in space			33 (13.8%)	8 (11.3%)	13 (15.3%)	
	Headache			6 (2.5%)	2 (2.8%)	2 (2.4%)	
	Disorientation or confusion			59 (24.7%)	20 (28.2%)	24 (28.2%)	
	Unable to keep your train of thought/inability to think clearly			7 (2.9%)	1 (1.4%)	3 (3.5%)	
	A feeling like you needed to jumpstart your brain			56 (23.4%)	17 (23.9%)	17 (20.0%)	
	Other			2 (0.8%)	0 (0%)	1 (1.2%)	
**Reasons for SHAAKEs because of a concussion (select all)**							.62
	Neck pain			34 (14.2%)	8 (11.3%)	13 (15.3%)	
	A feeling of a change in temperature or chills			13 (5.4%)	2 (2.8%)	6 (7.1%)	
	Emotional reaction to preceding event			76 (31.8%)	23 (32.4%)	17 (20.0%)	
	Pain to your face, scalp, or other part of your head that was not a headache			39 (16.3%)	14 (19.7%)	12 (14.1%)	
	Changes in your vision			101 (42.3%)	34 (47.9%)	40 (47.1%)	.96
			Double vision	27 (11.3%)	11 (15.5%)	12 (14.1%)	
			Blurred vision	72 (30.1%)	24 (33.8%)	27 (31.8%)	
			Trouble focusing	70 (29.3%)	25 (35.2%)	27 (31.8%)	
			Changes in colors perceived	13 (5.4%)	5 (7.0%)	6 (7.1%)	
			Slanted vision	10 (4.2%)	4 (5.6%)	3 (3.5%)	
			Other	11 (4.6%)	2 (2.8%)	3 (3.5%)	
	Auditory changes			47 (19.7%)	11 (15.5%)	25 (29.4%)	.95
			Ringing in your ears	41 (17.2%)	10 (14.1%)	22 (25.9%)	
			Deafness or impaired ability to hear sounds	15 (6.3%)	4 (5.6%)	9 (10.6%)	
			Trouble recognizing sounds	17 (7.1%)	5 (7.0%)	9 (10.6%)	
			Other	1 (0.4%)	0 (0.0%)	0 (0.0%)	
	Dizziness			105 (43.9%)	31 (43.7%)	44 (51.8%)	
	Impaired ability to balance			59 (24.7%)	19 (26.8%)	24 (28.2%)	
	Changes to your perception of space or perception of your body in space/proprioception			97 (40.6%)	24 (33.8%)	38 (44.7%)	
	Headache			68 (28.5%)	21 (29.6%)	27 (31.8%)	
	Disorientation or confusion			136 (56.9%)	42 (59.2%)	47 (55.3%)	
	Unable to keep your train of thought/inability to think clearly			68 (28.5%)	23 (32.4%)	26 (30.6%)	
	A feeling like you needed to jumpstart your brain			100 (41.8%)	30 (42.3%)	32 (37.6%)	
	Other			1 (0.4%)	0 (0.0%)	1 (1.2%)	

* *p*-values are provided to assess differences between soccer and football players. For categorical variables, chi-square was performed except where Fisher’s exact test was more appropriate. For continuous variables, Wilcoxon’s rank sum tests were performed because data were non-normally distributed. IQR: interquartile range; SHAAKE: spontaneous headshake after a kinematic event.

## Data Availability

The data that support the findings of this study are available from the corresponding author, D.H.D., upon reasonable request.

## Supplementary Materials

The following supporting information can be downloaded at: https://www.mdpi.com/article/10.3390/diagnostics14202314/s1. Table S1: Complete Survey; Table S2: Other reasons for shaking their head after an impact.
