# Supplementary material for: Spontaneous Headshake after a Kinematic Event (SHAAKE): Evaluating the Utility of a Potential New Sign in the Diagnosis of Concussion
Source: Diagnostics (Basel). 2024 Oct 17;14(20):2314. doi: 10.3390/diagnostics14202314 (PMC11506366; doi:10.3390/diagnostics14202314)
Supplement: Supplementary file 1 [file diagnostics-14-02314-s001.zip › diagnostics-3230161-supplementary.pdf]

Table S1 Complete Survey

Field Label	Choices, Calculations, OR Slider Labels	Field Note	Identifier?	Branching Logic (Show field only if...)
What is your age?				
What is your gender?	1, Male | 2, Female | 3, Non-binary | 4, Other			
Other gender:				[gender] = '4'
What is your race and ethnicity? (select all that apply)	1, American Indian or Alaska Native | 2, Asian | 3, Black or African American | 4, Hispanic or Latino or Spanish Origin | 5, Middle Eastern or North African | 6, Native Hawaiian or Other Pacific Islander | 7, White or Caucasian | 8, A race/ethnicity not listed here			
Other race/ethnicity not listed here:				[race(8)] = '1'
What was your highest level of sport participation?	1, Youth (typically less than 13 years old) | 2, High school/secondary school (typically between 13 and 18 years old) | 3, College (typically between 18 and 22 years old) | 4, Semi-professional | 5, Professional | 6, Other			
Other level:				[level] = '6'
What sport(s) do/did you play competitively? (select all that apply)	1, None | 2, Basketball | 3, Baseball | 4, Cheerleading | 5, Cross Country | 6, Cycling | 7, Diving | 8, Fencing | 9, Field Hockey | 10, Figure Skating | 11, Football (American) | 12, Football (Australian) | 13, Football (Soccer) | 14, Golf | 15, Gymnastics | 16, Ice Hockey | 17, Lacrosse | 18, Martial Arts | 19, Rugby | 20, Soccer | 21, Softball | 22, Swimming | 23, Tennis | 24, Track & Field | 25, Water Polo | 26, Wrestling | 27, Volleyball | 28, Other			
How many seasons of basketball did you compete in?				[sport(2)] = '1'
How many seasons of baseball did you compete in?				[sport(3)] = '1'
How many seasons of cheerleading did you compete in?				[sport(4)] = '1'
How many seasons of cross country did you compete in?				[sport(5)] = '1'
How many seasons of cycling did you compete in?				[sport(6)] = '1'
How many seasons of diving did you compete in?				[sport(7)] = '1'
How many seasons of fencing did you compete in?				[sport(8)] = '1'
How many seasons of field hockey did you compete in?				[sport(9)] = '1'
How many seasons of figure skating did you compete in?				[sport(10)] = '1'
How many seasons of American football did you compete in?				[sport(11)] = '1'
How many seasons of Australian football did you compete in?				[sport(12)] = '1'
How many seasons of soccer (international football) did you compete in?				[sport(13)] = '1' or [sport(20)] = '1'
How many seasons of golf did you compete in?				[sport(14)] = '1'
How many seasons of gymnastics did you compete in?				[sport(15)] = '1'
How many seasons of ice hockey did you compete in?				[sport(16)] = '1'
How many seasons of lacrosse did you compete in?				[sport(17)] = '1'
How many seasons of martial arts did you compete in?				[sport(18)] = '1'
How many seasons of rugby did you compete in?				[sport(19)] = '1'
How many seasons of softball did you compete in?				[sport(21)] = '1'
How many seasons of swimming did you compete in?				[sport(22)] = '1'

How many seasons of tennis did you compete in?		[sport(23)] = '1'
How many seasons of track and field did you compete in?		[sport(24)] = '1'
How many seasons of water polo did you compete in?		[sport(25)] = '1'
How many seasons of wrestling did you compete in?		[sport(26)] = '1'
How many seasons of volleyball did you compete in?		[sport(27)] = '1'
Other sport(s) you competed in:		[sport(28)] = '1'
How many seasons of other sports did you compete in?		[sport(28)] = '1'
Do/Did you serve in the military?		
How many years did you serve in the military?		[military] = '1'
Where do you live?	1, North America (Northern) | 2, North America (Central) | 3, North America (Carribean) | 4, South America | 5, Europe (Eastern) | 6, Europe (Northern) | 7, Europe (Southern) | 8, Europe (Western) | 9, Australia or New Zealand | 10, Africa (Eastern) | 11, Africa (Middle) | 12, Africa (Northern) | 13, Africa (Southern) | 14, Africa (Western) | 15, Asia (Central) | 16, Asia (Eastern) | 17, Asia (Middle East) | 18, Asia (Southern) | 19, Asia (Southeastern) | 20, Asia (Western) | 21, Melanesia, Micronesia, or Polynesia | 22, Other	
Other region		[region] = '22'
Where in Northern America do you live?	1, United States (Great Lakes) | 2, United States (Midwest) | 3, United States (New England) | 4, United States (Northwest) | 5, United States (South) | 6, United States (Southeast) | 7, United States (Southwest) | 8, Canada | 9, Bermuda | 10, Greenland | 11, Saint Pierre and Miquelon | 12, Other	[region] = '1'
Other Northern America region:		[region_america] = '12'
Sometimes a person will rapidly shake their head from side-to-side after a collision. Here are some examples of this movement.		
Do you remember ever making this type of head motion after a collision?		
What were the reasons you shook your head from side to side after a collision? (select all that apply)	1, Neck pain | 2, A feeling of a change in temperature or chills | 3, Emotional reaction to preceding event | 4, Pain to your face, scalp, or other part of your head that was not a headache | 5, Changes in your vision | 6, Auditory changes | 7, Dizziness | 8, Impaired ability to balance | 9, Changes to your perception of your body's positioning or location in space (proprioception) | 10, Headache | 11, Disorientation or confusion | 12, Unable to keep your train of thought / Inability to think clearly | 13, A feeling like you needed to jumpstart your brain | 14, Other	[shoc_yn] = '1'
Which vision changes: (select all that apply)	1, Double vision | 2, Blurred vision | 3, Trouble focusing | 4, Changes in colors perceived | 5, Slanted vision | 6, Other	[shoc_y(5)] = '1'
Other vision changes:		[shoc_y_vis(6)] = '1'
Which auditory changes: (select all that apply)	1, Ringing in your ears | 2, Deafness or impaired ability to hear sounds | 3, Trouble recognizing sounds | 4, Other	[shoc_y(6)] = '1'
Other auditory changes:		[shoc_y_aud(4)] = '1'
Other reasons you shook your head from side to side:		[shoc_y(14)] = '1'
Considering all the times that you rapidly shook your head	1, Neck pain | 2, A feeling of a change in temperature or chills | 3, Emotional reaction to preceding event | 4, Pain to your face, scalp, or other part of your	[shoc_yn] = '1'

after a collision, what was the most common reason?	head that was not a headache | 5, Changes in your vision | 6, Auditory changes | 7, Dizziness | 8, Impaired ability to balance | 9, Changes to your perception of space or perception of your body in space | 10, Headache | 11, Disorientation or confusion | 12, Unable to keep your train of thought / Inability to think clearly | 13, A feeling like you needed to jumpstart your brain | 14, Other	
Which vision changes:	1, Double vision | 2, Blurred vision | 3, Trouble focusing | 4, Changes in colors perceived | 5, Slanted vision | 6, Other	[shoc_y_most] = '5'
Other vision changes:		[shoc_y_most_vis] = '6'
Which auditory changes:	1, Ringing in your ears | 2, Deafness or impaired ability to hear sounds | 3, Trouble recognizing sounds | 4, Other	[shoc_y_most] = '6'
Other auditory changes:		[shoc_y_most_aud] = '4'
Other reasons you shook your head from side to side:		[shoc_y_most] = '14'
Approximately how many times do you estimate that you experienced a collision where you rapidly shook your head? (if you cannot remember, please provide your best guess)		[shoc_yn] = '1'
Some people have the misconception that concussions only happen when you black out after a hit to the head or when the symptoms last for a while. But, in reality, a concussion has occurred anytime you have had a blow to the head that caused you to have symptoms for any amount of time. These include: blurred or double vision, seeing stars, sensitivity to light or noise, headache, dizziness or balance problems, nausea, vomiting, trouble sleeping, fatigue, confusion, difficulty remembering, difficulty concentrating, or loss of consciousness. Whenever anyone gets a ding or their bell rung, that too is a concussion.		
Based on that definition, have you ever had a concussion?		
Based on that definition, how many times have you had a concussion? (if you cannot remember, please provide your best guess)		[conc_yn] = '1'
How many times have you been formally diagnosed with a concussion? (if you cannot remember, please provide your best guess)		[conc_yn] = '1'
Based on that definition, did you ever rapidly shake your head after a collision because of a concussion?		[conc_yn] = '1'
Of the [conc_num] concussions you experienced, approximately how many of those times did you rapidly shake your head afterwards? normal;">(if you cannot remember, please provide your best guess)		[shoc_bc_conc_yn] = '1'
Approximately how many of these [shoc_bc_conc_num] concussions (where you rapidly shook your head after the collision) were diagnosed? (if you cannot remember, please provide your best guess)		[shoc_bc_conc_yn] = '1'
For the [shoc_bc_conc_num] concussions where you rapidly shook your head afterwards, why did you rapidly shake your head? (select all that apply)	1, Neck pain | 2, A feeling of a change in temperature or chills | 3, Emotional reaction to preceding event | 4, Pain to your face, scalp, or other part of your head that was not a headache | 5, Changes in your vision | 6, Auditory changes | 7, Dizziness | 8, Impaired ability to balance | 9, Changes to your perception of space or perception of your body in space/proprioception (could overlap with vision) | 10, Headache | 11, Disorientation or confusion | 12, Unable to keep your train of thought / Inability to think clearly | 13, A feeling like you needed to jumpstart your brain | 14, Other	[shoc_bc_conc_yn] = '1'
Which vision changes: (select all that apply)	1, Double vision | 2, Blurred vision | 3, Trouble focusing | 4, Changes in colors perceived | 5, Slanted vision | 6, Other	[shoc_bc_conc_y(5)] = '1'

Other vision changes:		[shoc_bc_conc_y_vis(6)] = '1'
Which auditory changes: (select all that apply)	1, Ringing in your ears | 2, Deafness or impaired ability to hear sounds | 3, Trouble recognizing sounds | 4, Other	[shoc_bc_conc_y(6)] = '1'
Other auditory changes:		[shoc_bc_conc_y_aud(4)] = '1'
Other reasons you rapidly shook your head:		[shoc_bc_conc_y(14)] = '1'
	[shoc_num]-[shoc_bc_conc_num]	0=5
For the [shoc_no_conc_num] times you rapidly shook your head after a collision, but did not have a concussion, why did you rapidly shake your head?	1, Neck pain | 2, A feeling of a change in temperature or chills | 3, Emotional reaction to preceding event | 4, Pain to your face, scalp, or other part of your head that was not a headache | 5, Changes in your vision | 6, Auditory changes | 7, Dizziness | 8, Impaired ability to balance | 9, Changes to your perception of space or perception of your body in space/proprioception (could overlap with vision) | 10, Headache | 11, Disorientation or confusion | 12, Unable to keep your train of thought / Inability to think clearly | 13, A feeling like you needed to jumpstart your brain | 14, Other	[shoc_num] > [shoc_bc_conc_num] or ([shoc_yn] = '1' and [conc_yn] = '0')
Which vision changes: (select all that apply)	1, Double vision | 2, Blurred vision | 3, Trouble focusing | 4, Changes in colors perceived | 5, Slanted vision | 6, Other	[shoc_no_conc(5)] = '1'
Other changes to your vision:		[shoc_no_conc_vis(6)] = '1'
Which auditory changes: (select all that apply)	1, Ringing in your ears | 2, Deafness or impaired ability to hear sounds | 3, Trouble recognizing sounds | 4, Other	[shoc_no_conc(6)] = '1'
Other auditory changes:		[shoc_no_conc_aud(4)] = '1'
Other reasons you rapidly shook your head:		[shoc_no_conc(14)] = '1'
When was your most recent concussion?	1, Within the past week | 2, Between one week and one month ago | 3, Between one month and six months ago | 4, Between six months and one year ago | 5, Between one and five years ago | 6, Between five and ten years ago | 7, Between ten and twenty years ago | 8, More than twenty years ago	[conc_yn] = '1'
What percent of your concussions were caused by sports? (if you cannot remember, please provide your best guess)		[conc_yn] = '1'

Table S2 Other reasons for shaking their head after an impact

**Other Reasons Eight Respondents Reported that They Rapidly Shook Their Head after an Impact**
Cant remember
Faint, feeling like I was about to faint so I shook my head
Feels like I'm trying to shake off the headache or the "lights turned off" for a second
Just know something isn't right, like trying to shake everything back into place and shake the bad off
I would see what could be described as yellow lights in my vision whenever this happened and I'd feel really weird, floaty, almost good? I don't ever (in the moment) recall there being any significant pain from the impact, more like... Disorientation an instinctual desire to regain my senses? I would shake my head, sounds wouldn't be heard, I'd feel cold (like a chill down my spine) and eventually I would be able to perceive things like before the impact.
Remove sweat
Learned behavior
Compulsion to do so, unable to stop
